# Effectiveness of psychological therapies for depression and anxiety in adults with and without ADHD in England: a retrospective cohort study

**DOI:** 10.1016/j.lanepe.2026.101798

**Published:** 2026-08-06

**Authors:** Celine El Baou, Jae Won Suh, Rob Saunders, Joshua E.J. Buckman, Natasha Hickmott, Lucy Corrigan, Henry Clements, Dave Dagnan, Warren James Donnellan, William Mandy, Dilek Ocal, Aysha Mohamed Rafik Patel, Georgia Pavlopoulou, Elizabeth Pellicano, Steve Pilling, Ailsa Russell, Henry Shelford, Gavin Stewart, Joshua Stott, Amber John

**Affiliations:** a Clinical, Educational, and Health Psychology (CEHP), University College London, London, UK; b Kent and Medway NHS Talking Therapies, Canterbury, UK; c Department of Psychology, University of Liverpool, Liverpool, UK; d Cumbria, Northumberland, Tyne and Wear NHS Foundation Trust, Community Learning Disability Team, Workington, UK; e University of Bath, Bath, UK; f ADHD UK, London, UK; g Social, Genetic and Developmental Psychiatry Centre, Kings College London, London, UK

**Keywords:** ADHD, Depression, Anxiety, Interventions, Talking therapies

## Abstract

**Background:**

People with ADHD disproportionately experience depression and anxiety across the lifespan than people without. To date interventions have focused on managing ADHD. This study aimed to evaluate the effectiveness of therapies designed to alleviate depression and anxiety.

**Methods:**

We used linked electronic healthcare records of over 3 million adults over 18 years old who received a course of therapy in NHS Talking Therapies for depression and anxiety in England between 2012 and 2022. We identified a cohort of N = 13,693 adults with diagnosed ADHD and calculated pre-post effect sizes (Cohen’s d_av_). We then identified a propensity-matched comparison group of N = 13,693 adults without diagnosed ADHD, and conducted logistic regressions to compare outcomes between the two groups; and ran exploratory subgroup analyses to evaluate whether known associations between therapy outcomes and socio-demographic factors were modified by ADHD diagnosis status.

**Findings:**

On average, people with ADHD experienced moderate-to-large reductions in their depression (pre-post effect size Cohen’s d_av_ = −0.72 [95% CI −0.75 to −0.70]) and anxiety (Cohen’s dav = −0.72 [95% CI −0.74 to −0.69]) symptoms between before, and after therapy. People with ADHD had poorer therapy outcomes, regardless of matching and adjustment for covariates (Reliable improvement from depression and anxiety symptoms: OR = 0.77, 95% CI 0.73–0.81, Reliable recovery: OR = 0.69 95% CI 0.65–0.72, Reliable deterioration: OR = 1.38, 95% CI 1.25–1.50). There was no strong evidence that associations between therapy outcomes and socio-demographic factors were affected by ADHD status, but small sample sizes limited interpretations for older people and people from minority ethnic groups.

**Interpretation:**

People with ADHD may benefit from interventions designed to improve their mental health as opposed to managing ADHD. There is a need for evidence-based guidelines to adapt mental health interventions for this group, and a better understanding of how systemic barriers to care and diagnosis may affect outcomes.

**Funding:**

Medical Research Foundation (MRF).

**Research in context**

**Evidence before this study**

Adults with ADHD are disproportionally affected by depression and anxiety compared to the general population, potentially because of common neurobiological mechanisms associated with these conditions or an accumulation of social risks across the lifespan. Psychological interventions for this population have focused on ADHD management and have not been designed specifically to alleviate depression or anxiety. Treatment guidelines recommend that adults with ADHD experiencing depression or anxiety are referred to non-specialised mental health services, but evidence is lacking with regards to the effectiveness of interventions provided in such settings for people with ADHD. We searched PubMed for studies in English published from database inception to 12 January 2026, using all fields: (anxi∗ OR depress∗) AND (“psycho∗ therap∗” OR “mental health interven∗”) AND (ADHD OR “attention deficit∗” OR “attention deficit and hyperactivity disorder”). Out of 103 search results, one feasibility, single-arm study evaluated a digital mental health intervention in 30 adults with ADHD experiencing co-occurring depression and anxiety.

**Added value of this study**

This study built on existing findings from one feasibility single-arm trial. Using a cohort of over 13,000 adults with a formal diagnosis of ADHD, we found that adults with ADHD can benefit from psychological therapies offered in non-specialised settings to improve their mental health, but that their outcomes are poorer than outcomes in a cohort of people without diagnosed ADHD matched on similar characteristics. Our study is, to our knowledge the first large scale study to show that adults with diagnosed ADHD experiencing co-occurring depression and anxiety may benefit from talking therapies provided in non-specialist care settings, but also that their therapy outcomes are poorer than a comparison group of people without diagnosed ADHD.

**Implications of all the available evidence**

Our findings have important clinical implications, since they show the potential benefit of interventions targeting mental health symptoms rather than ADHD traits to improve wellbeing of adults with ADHD. Our findings also show that therapies provided in routine care settings are sub-optimal for people with ADHD, we suggest that further research should identify how such therapies may be adapted to improve outcomes and accessibility to mental health care for adults with ADHD.

## Introduction

Attention deficit hyperactivity disorder (ADHD) has been conceptualised as a neurodevelopmental condition, characterised by traits of inattention, hyperactivity, and impulsivity. These traits typically occur across multiple settings and may interfere with daily life. Research informed by lived experience has also highlighted positive attributes, including high energy, creativity, curiosity, and the capacity for hyperfocus, which may be advantageous in some contexts.^1^ ADHD persists into adulthood for many people, with an estimated point-prevalence of 3.1%.^2^ Despite this, it remains underdiagnosed in adults, particularly among marginalised groups such as people from ethnic minority groups, women, and older adults.^3^ In this study, ADHD is framed as a condition that interacts with its wider psychosocial context, we acknowledge and respect the diversity of preferences when it comes to language use and conceptualisation around ADHD.^4^

Evidence suggests that challenges people with ADHD face are not solely intrinsic but reflect the interaction between ADHD traits and environments that fail to accommodate them. From childhood, people with ADHD face heightened experiences of social exclusion. Such inequalities frequently persist into adulthood since adults with ADHD are also disproportionately affected by adverse health outcomes, homelessness, increased suicidality and shorter life expectancy.^5^

Consequently, ADHD is associated with much higher point-prevalence rates of depression (8.6%–55% compared with 1.2%–12.5% in the general population) and anxiety disorders (4.3%–47.1% compared with 0.5%–9.5%).^6^ This high prevalence may reflect specific neurobiological mechanisms,^7^ or the accumulation of social risk across the lifespan highlighted previously. This makes access to timely, evidence-based, and effective mental health interventions critical to improve wellbeing and quality of life.

However, there is a lack of evidence with regards to the effectiveness psychological therapies typically used in the general population when applied to people with ADHD. Cognitive and Behavioural Therapy (CBT) offered to manage ADHD core difficulties may also be helpful in alleviating anxiety and depression symptoms, but interventions were designed for ADHD management, and conducted in people with ADHD regardless of whether they experience depression or anxiety at a level that warrants a clinical intervention. One study including adults with ADHD experiencing co-occurring depression and anxiety, showed good improvement in depression and anxiety symptoms after a CBT and mindfulness based digital mental health intervention, but included just 30 adults and did not include a control group.^8^ Consequently, to our knowledge there are currently no studies evaluating whether evidence-based psychological therapies offered in non-ADHD-specialist services are also effective for people with ADHD. Addressing this is critical because evidence-based psychological therapies, such as CBT are recommended as a first-line treatment for depression and anxiety, including for people with ADHD.^9^

In England, evidence-based psychological therapies for adults with depression or anxiety are typically provided in the National Health Service (NHS) primary and community care psychological therapy programme called NHS Talking Therapies for anxiety and depression (NHS TTad). In England, treatment guidelines recommend CBT for those people with ADHD who do not respond to medication, or decide to not take medication. In reality, barriers to ADHD and assessment and treatments are such that the majority of people with ADHD will not receive psychological therapy for ADHD.^10^ On the contrary, NHS TTad services, that are not designed to support people with ADHD, are widely available, and people with ADHD who experience depression, anxiety, or both, will be referred to NHS TTad. There is currently no national guidance to support clinicians on which adaptations may be beneficial to people with ADHD in these non-specialist services. Consequently, it is important to establish whether interventions provided in NHS TTad are effective for people with ADHD. Moreover, there are known associations between therapy outcomes and socio-demographic factors, such as age, gender, ethnicity, social deprivation, taking psychotropic medications and long-term health conditions,^11^, ^12^, ^13^, ^14^, ^15^ and it is not known whether such associations may be modified by the presence of an ADHD diagnosis.

In line with guidance for evaluating complex interventions from the medical research council (MRC),^16^ this study uses a naturalistic design to evaluate psychological therapy outcomes for adults with ADHD attending NHS TTad services across England. This study evaluated three following research questions. The first evaluated whether adults with ADHD experience a reduction in their depression or anxiety symptoms after a course of psychological therapy. The second examined whether psychological therapy outcomes differ between people with ADHD and people without identified ADHD. The third explored whether known associations between socio-demographic factors and therapy outcomes are modified by the presence of an ADHD diagnosis.

## Methods

This study followed the Enhancing the Quality and Transparency of Health Research (EQUATOR) reporting guidelines: REporting of studies Conducted using Observational Routinely-collected health Data (RECORD)^17^ (Appendix p21).

### Data

This study used the MODIFY dataset.^18^ We utilised electronic healthcare records from people who received psychological therapy in NHS TTad between the 1st April 2012 and 31st March 2022.^19^ The NHS TTad dataset does not include information about ADHD status. To obtain this, records were linked with databases where this information is available: the Hospital Episode Statistics (HES) dataset; the Mental Health Services Dataset (MHSDS); and HES-ONS mortality dataset, using a key provided by NHS Digital. The MODIFY dataset includes information on demographic characteristics and psychological therapy factors (e.g., number of sessions) for each person (Appendix p2).

### Intervention

To access psychological therapy in NHS TTad, people can either self-refer or be referred by any health professional. The type of treatment is chosen to follow clinical guidelines from the National Institute of Health and Care Excellence (NICE), which are based on the extant research evidence. Treatment protocols are designed to address difficulties associated with common mental health problems, rather than specific difficulties associated with core ADHD traits. Therapy within services follows a stepped-care model: depending on the presentation, people may initially be offered a “low intensity” intervention (for example: up-to six sessions of CBT-based guided self-help) or a “high intensity” intervention (which typically includes a longer course of 1:1 therapy, e.g., CBT or counselling). It is possible clinicians may adapt therapy depending on their own awareness of the condition or guidance provided at service level. Treatment ends either through completion of the modules or sessions, through mutual agreement between the clinician and the person receiving the intervention, or when the person decides they no longer want to pursue the intervention.

### Study cohort

A study cohort was formed using the linked-data following convention from similar studies,^18^ including all adults who completed a course of psychological therapy (a minimum of one assessment and one treatment session) in NHS TTad according to NHS established evaluation criteria^19^ and have both a record in NHS TTad and a linked record in the HES/MHSDS databases.

**Fig. 1:**
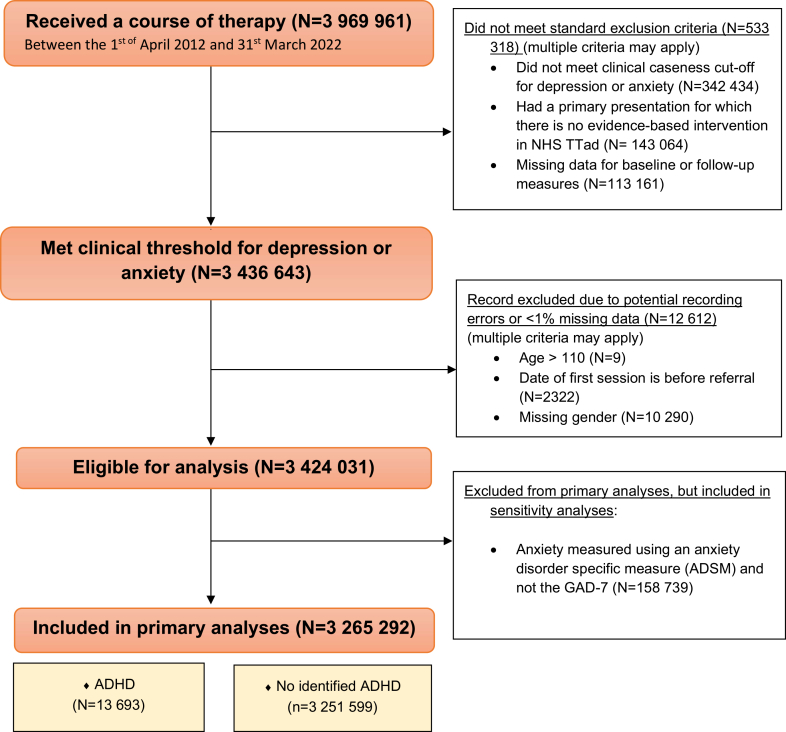
**Study flow chart.** Abbreviations: NHS TTad = NHS Talking Therapies for anxiety and depression; ADHD = Attention Deficit Hyperactivity Disorder; GAD = Generalised anxiety disorder; ADSM = Anxiety Disorder Specific Measure.

For people who had more than one episode of care in an NHS TTad service during the study period, only data for the first course of treatment were used. A standard set of exclusion criteria used in studies using NHS TTad cohorts were applied^18^ (see Fig. 1 for a study flow chart). Missing data for depression and anxiety measures accounted for less than 1% of the sample and were excluded (N = 113,161, N = 40,498 pre therapy, N = 100,418 post-therapy) since previous research has found the impact of excluding such cases to be negligible.^20^

For a comparatively small group of individuals (N = 158,739, 4.6% of the total study cohort), Anxiety Disorder Specific Measures (ADSM) were used to measure improvement in anxiety outcomes in place of the GAD-7. For consistency, these records were excluded from the primary analyses but included in sensitivity analyses.

The final study cohort of 3,265,292 individuals eligible for analysis, included 13,693 (0.41%) adults with identified ADHD. See Fig. 1 for a study flowchart.

#### Exposure

ADHD status was identified using diagnostic codes entered in the HES and MHSDS databases, according to the International Classification of Diseases, 10th Revision (ICD-10) using codes F90.0, F90.1, F90.2, F90.8, F90.9 as per previous research.^21^ To reflect that ADHD persists over the lifespan, diagnosis codes were considered regardless of their temporality to NHS TTad referral. A sensitivity analysis was also conducted by defining the ADHD cohort as people whose first occurrence of an ADHD diagnosis code was before NHS TTad referral.

#### Outcome measures

Outcomes were nationally defined outcome metrics for NHS TTad, derived from depression and anxiety measures collected in services before, and after therapy. Thresholds used to refer to symptoms likely to be sufficient for meeting diagnostic criteria (referred to as “caseness thresholds”) for depression and anxiety were defined as in previous research and have been used in this study to mirror the thresholds used in practice in NHS TTad services.^22^ Depression was assessed using the PHQ-9^23^ with a caseness threshold score ≥10. Generalised anxiety was assessed using the GAD-7, with a caseness threshold score of ≥8.^24^ The PHQ-9 and GAD-7 have both been used with populations of adults with ADHD, but their psychometric properties in this population are to be fully validated.^25^^,^^26^

For the first research question about pre-post changes in people with ADHD, outcomes were pre-post changes on measures of depression (PHQ-9), and generalised anxiety (GAD-7).

For the second research question, primary outcomes were nationally defined outcome metrics for NHS TTad were derived from the symptom measures above.^22^ *Reliable improvement* corresponds to a reduction in depression or anxiety symptoms from the first to last attended treatment session that exceeds the threshold for error of measurement on either symptom scale (≥6 points on PHQ-9 or ≥4 points on GAD-7), without a reliable deterioration (defined below) on either symptom scale. *Recovery* is defined as ending treatment below the threshold for ‘caseness’ on both the measures of depression and anxiety. Assessing “*Recovery*” is an intermediate step in the definition of “reliable recovery”. *Reliable recovery* corresponds to reliable improvement plus “*recovery*”. *Reliable deterioration* is an increase in depression or anxiety symptoms from the first to last attended treatment session by at least the magnitude of the threshold for the error of measurement (see reliable improvement above), and no reliable decrease.

For this second research question, pre-post change on measures of depression (PHQ-9), and generalised anxiety (GAD-7) were secondary outcomes.

For the third exploratory research question, outcomes were reliable improvement, reliable recovery and reliable deterioration.

#### Covariates

A range of covariates measured at referral or assessment and known to be associated with therapy outcomes were included in analyses, including age, gender, ethnicity, employment status, taking psychotropic medication (for mental health) prior to starting therapy, self-report of a long-term health condition. Analyses were additionally adjusted for factors modifiable by services: number of high and low intensity therapy sessions, reason for ending therapy, frequency of sessions, waiting times (see Appendix p5 for more details).

### Statistical analysis

Analyses were conducted using Stata 18.^27^ First, we calculated summary statistics of demographic characteristics and treatment factors for adults with and without a diagnosis of ADHD. Missing data for categorical variables were coded as a “missing” category. To contextualise the generalisability of study findings, we also calculated the expected prevalence of ADHD in a population of people with depression or anxiety^6^ and compared it to the prevalence of ADHD in the cohort. Next, for the first research question, pre-post treatment differences in depression and anxiety symptoms for the ADHD group were computed, using Cohen’s d measure of effect size. In the absence of availability of a comparison group of people with ADHD who may receive a different intervention, to contextualise our results, we gathered data from RCTs including people with ADHD who received psychological therapy for ADHD, and included depression or anxiety as outcomes, and calculated a pre-post Cohen’s d_av_.^28^ For the second research question, we investigated whether a diagnosis of ADHD (vs. no diagnosis) was associated with therapy outcomes. For the primary outcomes, logistic regressions were conducted despite the variety of treatment durations in the cohort, to reflect clinical practice and national metrics based on outcomes at the end of therapy. Models using binary outcome measures as the dependent variable were run in the following sequence: 1) including group (Identified ADHD vs. no identified ADHD); 2) adjusted for clinical and socio-demographic covariates, 3) additionally adjusted for NHS TTad therapy factors and 4) rerun using a propensity-score^29^ matched sample to further account for differences between the ADHD and comparison group, and as recommended when the size of the comparison group exceeds that of the treatment group.^30^ In this last analysis, adults with an ADHD diagnosis were matched with a person without identified ADHD using *psmatch2*.^31^ The propensity score was estimated using logistic regression including all factors possibly associated with the outcomes as covariates as described in Appendix p7. Exact matching was used for categorical covariates, with a caliper set to 0.1 for propensity score matching. When propensity score matching was used, matching was done without replacement. Model 4, the most adjusted after matching, was considered the primary model. For secondary outcomes, linear regressions were used examining pre-post changes in depression and anxiety symptoms as the dependent variable, adjusting for covariates in the same sequence. For the third research question, we investigated whether covariates listed previously were associated with different therapy outcomes for people with ADHD vs. the comparison group. This was achieved using Wald tests for the presence of an interaction between belonging to the ADHD group and each demographic or clinical factor in the most adjusted models in both the matched and full cohort, for primary outcomes. Given that p-values may be sensitive to large sample sizes,^32^ interaction tests results were evaluated jointly with adjusted outcomes within subgroups. Finally, sensitivity analyses were conducted by re-running the analyses: 1) including those records where ADSM measures were used for anxiety symptoms, 2) by defining the ADHD cohort as people having a first occurrence of a diagnosis code before NHS TTad and 3) including NHS Clinical Commissioning Group (CCG) as a categorical covariate, to evaluate whether analyses are affected by service-level disparities.

### Involvement of people with lived experience in the conduct of the study

The research team who designed and conducted this study includes co-authors with lived of experience of ADHD, neurodivergence, and/or of attending NHS TTad services as a user. To ensure that this research reflects the experience of the wider ADHD community, a person with lived experience from ADHD UK also provided input into the analyses and write-up of this manuscript. Lived experience input influenced this manuscript in the depiction of a nuanced report of the lived experience of ADHD and its interaction with co-occurring mental health challenges and systemic stressors.

### Ethics

Non-identifiable information was provided by NHS Digital with a legal basis for the anonymization, meaning that this research did not require informed consent. Ethical approval for this research was granted by the UCL department of Clinical, Educational and Health Psychology ethics committee (CEHP/2023/592B).

### Role of the funding source

The funders of the study were not involved in the study design, data analysis, interpretation of data, writing of the report, and in the decision to submit the paper for publication.

## Results

The study cohort included 3,265,292 people who received a course of therapy in NHS TTad between the 1st April 2012 and the 31st March 2022. A total of N = 13,693 people with a diagnosis code of ADHD were identified, compared to 3,251,599 people without a diagnosis code of ADHD, which corresponds to a prevalence of ADHD of 0.41% in the cohort. Based on data from prevalence studies^6^ we calculated that the prevalence of diagnosed ADHD could range between 12.3% and 18.7% in a cohort of people with depression, and 13.7% and 21.6% in a cohort of people with anxiety. The prevalence of ADHD was lower than expected in our cohort.

People with ADHD were on average younger and more likely to be male (mean age = 28.9 years old 6981 [51.0%] of 13,693 male) than people without a diagnosis code of ADHD (mean age = 39.5 years old, 1,089,527 [33.5%] of 3,251,599 were male). People with ADHD were less likely to belong to the Asian ethnic group, more likely to live in some of the most deprived areas of England, less likely to be employed and more likely to take psychotropic medications for depression or anxiety. A similar proportion of people with and without ADHD received therapy for depression as the main presenting problem, but people with ADHD were more likely to receive therapy for post-traumatic stress disorder than the comparison group and were less likely to receive therapy for generalised anxiety disorder (see Table 1).

**Table 1:** Demographics and baseline characteristics.

Demographic and baseline measures	Before matching		After matching	
	ADHD diagnosis code	No identified ADHD	ADHD diagnosis code	No identified ADHD
	N = 13,693	N = 3,251,599	N = 13,686	N = 13,686
**Demographics**				

Age at referral—Mean (SD) range	28.9 (9.8) 18–82	39.5 (14.9) 18–101	28.9 (9.8) 18–82	30.2 (10.7) 18–94
	n (%)	n (%)	n (%)	n (%)

Age category				
18–24	5808 (42.42%)	582,226 (17.91%)	5803 (42.40%)	5803 (42.40%)
25–44	6687 (48.84%)	1,521,150 (46.78%)	6685 (48.85%)	6685 (48.85%)
45–64	1141 (8.33%)	944,241 (29.04%)	1141 (8.34%)	1141 (8.34%)
65+	57 (0.42%)	203,982 (6.27%)	57 (0.42%)	57 (0.42%)
Ethnicity				
White	11,338 (82.80%)	2,624,444 (80.71%)	11,338 (82.84%)	11,338 (82.84%)
Mixed	472 (3.45%)	72,496 (2.23%)	471 (3.44%)	471 (3.44%)
Asian	279 (2.04%)	167,398 (5.15%)	278 (2.03%)	278 (2.03%)
Black	235 (1.72%)	91,819 (2.82%)	234 (1.71%)	234 (1.71%)
Other	165 (1.20%)	42,738 (1.31%)	163 (1.19%)	163 (1.19%)
Missing	1204 (8.79%)	252,704 (7.77%)	1202 (8.78%)	1202 (8.78%)
Gender				
Male	6981 (50.98%)	1,089,527 (33.51%)	6978 (50.99%)	6978 (50.99%)
Female	6712 (49.02%)	2,162,072 (66.49%)	6708 (49.01%)	6708 (49.01%)
IMD quintile				
1 (Most deprived)	3905 (28.52%)	703,732 (21.64%)	3904 (28.53%)	3904 (28.53%)
2	3155 (23.04%)	705,469 (21.70%)	3155 (23.05%)	3155 (23.05%)
3	3553 (17.83%)	642,187 (19.75%)	2438 (17.81%)	2438 (17.81%)
4	2041 (14.91%)	588,657 (18.10%)	2041 (14.91%)	2041 (14.91%)
5 (Least deprived)	1796 (13.12%)	531,627 (16.35%)	1794 (13.11%)	1794 (13.11%)
Missing	354 (2.59%)	79,927 (2.46%)	354 (2.59%)	354 (2.59%)
Employment status before therapy				
Employed	5430 (39.66%)	1,795,334 (55.21%)	5430 (69.68%)	5430 (69.68%)
Unemployed, seeking work	2918 (21.31%)	338,181 (10.40%)	2918 (21.32%)	2918 (21.32%)
Unemployed, not seeking work	2487 (18.16%)	625,117 (19.22%)	2487 (18.17%)	2487 (18.17%)
Long-term illness/benefits	1698 (12.40%)	212,881 (6.55%)	1692 (12.36%)	1692 (12.36%)
Missing	1160 (8.47%)	280,086 (8.61%)	1159 (8.47%)	1159 (8.47%)

Clinical measures pre-treatment, pre-existing conditions and medication				
	Mean (SD) range	Mean (SD) range	Mean (SD) range	Mean (SD) range
Depression symptoms pre-treatment (PHQ-9)	17.37 (5.15)	15.67 (5.49)	17.37 (5.15)	17.42 (5.22)
Anxiety symptoms pre-treatment (GAD-7)	15.26 (4.30)	14.24 (4.37)	15.26 (4.30)	15.29 (4.18)
	n (%)	n (%)	n (%)	n (%)

Taking psychotropic medication				
No	5453 (39.83%)	1,496,802 (46.04%)	5453 (39.83%)	5453 (39.83%)
Yes	6876 (50.22%)	1,441,233 (44.32%)	6876 (50.22%)	6876 (50.22%)
Missing	1364 (9.96%)	313,464 (9.64%)	1360 (9.93%)	1360 (9.93%)
Long-term health conditions (as self-reported in NHS TTad)				
No	5915 (43.20%)	1,487,512 (45.75%)	5914 (43.21%)	5914 (43.21%)
Yes	3442 (25.14%)	744,199 (22.89%)	3438 (25.12%)	3438 (25.12%)
Missing	4336 (31.67%)	1,019,888 (31.37%)	4334 (31.67%)	4334 (31.67%)
Co-occurring Autism (as per ICD-10 code)	2493 (18.21%)	13,380 (0.41%)		

NHS TTad treatment factors				
Presentation (as assessed in NHS TTad)				
Depression	4677 (34.16%)	1,149,267 (35.34%)	4675 (34.16%)	4771 (34.86%)
Mixed anxiety and depressive disorder	1773 (12.95%)	405,827 (12.48%)	1772 (12.95%)	1789 (13.07%)
GAD	2345 (17.13%)	696,799 (21.43%)	2344 (17.13%)	2310 (16.88%)
OCD	220 (1.61%)	35,675 (1.10%)	220 (1.61%)	227 (1.66%)
PTSD	442 (3.23%)	69,189 (2.13%)	442 (3.23%)	437 (3.19%)
Phobic anxiety & panic	683 (4.99%)	176,045 (5.41%)	682 (4.98%)	704 (5.14%)
Other anxiety disorder	147 (1.07%)	45,146 (1.39%)	147 (1.07%)	134 (0.98%)
Missing	3406 (24.87%)	673,651 (20.72%)	3404 (24.87%)	3314 (24.21%)
Year of referral				
2012	996 (7.27%)	230,196 (7.08%)	995 (7.27%)	1091 (7.97%)
2013	1339 (9.78%)	310,497 (9.55%)	1339 (9.78%)	1341 (9.80%)
2014	1586 (11.58%)	345,643 (10.63%)	1585 (11.58%)	1512 (11.05%)
2015	1616 (11.80%)	360,103 (11.07%)	1615 (11.80%)	1581 (11.55%)
2016	1442 (10.53%)	351,418 (10.81%)	1442 (10.54%)	1426 (10.42%)
2017	1380 (10.08%)	334,462 (10.29%)	1380 (10.08%)	1358 (9.92%)
2018	1381 (10.09%)	348,807 (10.73%)	1380 (10.08%)	1412 (10.32%)
2019	1318 (9.63%)	326,286 (10.03%)	1316 (9.62%)	1339 (9.78%)
2020	1414 (10.33%)	310,418 (9.55%)	1413 (10.32%)	1292 (9.44%)
2021	1221 (8.92%)	333,769 (10.26%)	1221 (8.92%)	1334 (9.75%)
Reason for ending therapy				
Completed	5780 (42.21%)	1,818,588 (55.93%)	5779 (42.22%)	5674 (41.45%)
Deceased	20 (0.15%)	6608 (0.17%)	21 (0.15%)	20 (0.15%)
Unscheduled discontinuation	4179 (30.52%)	792,340 (24.37%)	4177 (30.51%)	4291 (31.35%)
Mutual agreement or signposting	229 (1.67%)	54,813 (1.69%)	229 (1.67%)	230 (1.68%)
Referred on or Service not suitable	1380 (10.08%)	152,455 (4.69%)	4177 (30.51%)	4291 (31.35%)
Missing	2104 (15.37%)	427,896 (13.16%)	2104 (15.37%)	2152 (15.72%)
	Mean (SD) range	Mean (SD) range	Mean (SD) range	Mean (SD) range

Number of sessions^a^	6.19 (4.5) 2–23	6.51 (4.3) 2–23	6.20 (4.4) 2–23	6.27 (4.5) 2–23
Time between referral and assessment (weeks)^a^	4.94 (3.4) 0–30	3.98 (5.4) 0–30	4.94 (6.4) 0–30	4.88 (6.5) 0–30
Time between assessment and treatment (weeks)^a^	11.7 (12.90) 0–39	9.90 (11.5)	11.7 (12.9) 0–39	11.65 (12.8) 0–39
Average frequency of sessions (every x week)	3.03 (2.2) 0.14–11	2.84 (1.9) 0.14–11	3.04 (2.2) 0.14–11	3.04 (2.2) 0.14–11
	n (%)	n (%)	n (%)	n (%)

% with 6 low intensity sessions or more	1020 (7.45%)	348,615 (10.72%)	1020 (7.45%)	890 (6.50%)
% with 6 high intensity sessions or more	3017 (22.03%)	694,077 (21.35%)	3016 (22.04%)	3110 (22.72%)

IMD = Index of multiple deprivation, GAD = Generalised anxiety disorder, OCD = Obsessive compulsive disorder, PTSD = Post traumatic stress disorder, PHQ = Patient health questionnaire, SD = Standard Deviation.

a To reduce the influence of extreme values, variables were winsorized at the top 99% percentile.

On average, people with ADHD had slightly elevated depression and anxiety scores before starting therapy than people without. The ADHD group had slightly longer waiting times but was also more likely to receive high intensity treatment in the first instance (ADHD Group: 49.1%, Comparison Group: 41.9%). People in the ADHD group were more likely to discontinue therapy early (ADHD Group: 30.6%, Comparison Group: 24.3%), more likely to be referred on to other services, or for NHS TTad services to be deemed not suitable to support them (ADHD group: 10.1%, Comparison Group: 4.7%).

The propensity score matching algorithm identified a comparison observation for 13,686 people in the ADHD group, meaning a match was not found for 7 people in the ADHD group. After matching, demographic characteristics and therapy factors were similar in both the ADHD and comparison groups (Table 1).

In the pre-post analyses, people with ADHD experienced moderate to large reductions in both their depression (pre-post Cohen’s dav = −0.72 (95% CI −0.75 to −0.70) and anxiety (pre-post Cohen’s dav = −0.72 (95% CI −0.74 to −0.69) symptoms between before and after therapy (Table 2). This was larger or comparable to symptoms changes observed in RCTs for both depression (effect sizes ranging from dav = 0.02 [95% CI −0.69 to 0.74] to −0.83 [95% CI −1.36 to −0.3]), and anxiety (effect sizes ranging from dav = 0.35 [95% CI −0.39 to 1.1] to −0.79 [95% CI −1.34 to −0.25])^33^^,^^34^ (Appendix p10).

**Table 2:** Clinical measures and outcomes.

	Before matching		After matching	
	ADHD diagnosis code	No identified ADHD	ADHD diagnosis code	No identified ADHD
	N = 13,693	N = 3,251,599	N = 13,686	N = 13,686
Primary outcomes	n (%)	n (%)	n (%)	n (%)

Reliable improvement	7845 (57.29%)	2,266,524 (69.70%)	7842 (57.30%)	8698 (63.55%)
Recovery	4236 (30.94%)	1,622,845 (49.91%)	4234 (30.94%)	5057 (36.95%)
Reliable recovery	3991 (29.15%)	1,542,252 (47.43%)	3989 (29.15%)	4856 (35.48%)
Reliable deterioration	1239 (9.05%)	187,871 (5.78%)	1239 (9.05%)	854 (6.24%)
Secondary outcomes	Mean (SD)	Mean (SD)	Mean (SD)	Mean (SD)

PHQ9-baseline	17.37 (5.15)	15.67 (5.49)	17.37 (5.15)	17.42 (5.22)
PHQ9-After treatment	12.58 (6.95)	9.44 (6.68)	12.57 (6.94)	11.57 (7.08)
PHQ-9 change	4.79 (6.63)	6.23 (6.45)	4.79 (6.63)	5.85 (6.66)
GAD7- baseline	15.26 (4.30)	14.24 (4.37)	15.26 (4.30)	15.28 (4.19)
GAD7- after treatment	11.07 (6.04)	8.51 (5.81)	11.07 (6.04)	10.19 (6.05)
GAD7 change	4.19 (5.84)	5.72 (5.82)	5.10 (5.84)	5.89 (4.19)

n = Data available; SD = Standard Deviation; PHQ = Patient Health Questionnaire; GAD = Generalised Anxiety Disorder.

In the comparative analyses, however, their therapy outcomes were poorer than those of people without ADHD, regardless of adjustment for covariates and propensity-score matching. In unadjusted analyses, people with ADHD had 42% lower odds of reaching reliable improvement (57.3% vs. 69.70% OR = 0.58, 95% CI 0.56–0.60, p < 0.0001) and 54% lower odds of reaching reliable recovery (30.9% vs. 49.9% OR = 0.46, 95% CI 0.44–0.47, p < 0.0001) from their symptoms than people in the comparison group. They also had 62% higher odds of experiencing a reliable deterioration in symptoms (9.1% vs. 5.8% OR = 1.62, 95% CI 1.53–1.72, p < 0.0001).

Differences between the ADHD and comparison group were attenuated after adjusting for covariates and propensity score matching but remained statistically and clinically relevant for all outcomes. After propensity score matching, the odds of reaching reliable improvement and reliable recovery were respectively 26% lower (OR = 0.74, 95% CI 0.71–0.78, p < 0.0001) and 31% lower (OR = 0.69 95% CI 0.66–0.74, p < 0.0001) for people with ADHD, while their odds of reliable deterioration were 52% higher than for the comparison group (OR = 1.52, 95% CI 1.38–1.67, p < 0.0001). Similarly, people with ADHD experienced a smaller change in the PHQ-9 (LSMeans difference: −1.43, 95% CI −1.55 to −1.33, p < 0.0001) and GAD-7 (LSMeans difference: −1.36, 95% CI −1.46 to −1.26, p < 0.0001) score between before, and after therapy; differences that were attenuated after matching (LSMeans PHQ-9: −0.96, 95% CI −1.09 to −0.82, p < 0.0001, LSMeans GAD-7: −0.96, 95% CI −1.09 to −0.83, p < 0.0001) (Table 3).

**Table 3:** Study outcomes—logistic regression.

Primary outcomes	Reliable improvement				Reliable recovery				Reliable deterioration			
	N	OR	95% CI	p-value	N	OR	95% CI	p-value	N	OR	95% CI	p-value
Model 1–Full sample (unadjusted)	3,265,292	0.58	0.56–0.60	<.0001	3,265,292	0.46	0.44–0.47	<0.0001	3,265,292	1.62	1.53–1.72	<0.0001
Model 2–Full sample (adjusted)^a^	3,265,292	0.70	0.68–0.73	<0.0001	3,265,292	0.65	0.63–0.67	<0.0001	3,265,292	1.54	1.45–1.63	<0.0001
Model 3–Full sample (adjusted)^b^	3,265,292	0.77	0.75–0.80	<0.0001	3,265,292	0.70	0.68–0.74	<0.0001	3,265,292	1.39	1.31–1.48	<0.0001
Model 4–PS matched (adjusted)^c^	27,378	0.77	0.73–0.81	<0.0001	27,378	0.69	0.65–0.72	<0.0001	27,378	1.38	1.25–1.50	<0.0001

Secondary outcomes	PHQ-9 change				GAD-7 change			
	N	LS means difference 95% CI		p-value	N	LS means difference 95% CI		p-value
Model 1–Full sample (unadjusted)	3,265,292	−1.43	−1.55 to −1.33	<0.0001	3,265,292	−1.36	−1.46 to −1.26	<0.0001
Model 2–Full sample (adjusted)^a^	3,265,292	−1.36	−1.46 to −1.26	<0.0001	3,265,292	−1.17	−1.26 to −1.09	<0.0001
Model 3–Full sample (adjusted)^b^	3,265,292	−0.92	−1.00 to −0.83	<0.0001	3,265,292	−0.80	−0.87 to −0.71	<0.0001
Model 4–PS matched (adjusted)^c^	23,378	−0.96	−1.09 to −0.82	<0.0001	23,378	−0.96	−1.09 to −0.83	<0.0001

OR = Odds ratio, CI = Confidence Interval, PHQ = Patient Health Questionnaire, GAD = General Anxiety Disorder, PS = Propensity Score.

Missing data for categorical covariates was imputed by using a “missing” category. There was no missing data for continuous covariates.

a Adjusted for gender, ethnicity, employment status, LTC case, psychotropic medication, diagnosis category, IMD quintile, year of first appointment, age at referral, baseline PHQ-9, baseline GAD-7.

b Adjusted for gender, ethnicity, employment status, LTC case, psychotropic medication, diagnosis category, IMD quintile, year of first appointment, age at referral, baseline PHQ-9, baseline GAD-7, waiting times referral to assessment, waiting time assessment, year of appointment, number of low intensity sessions, number of high intensity sessions, reason for ending treatment, frequency of sessions.

c The propensity score was estimated using logistic regression using age group, gender, ethnicity, employment status, LTC case, psychotropic medication, IMD quintile as categorical covariates and year of first appointment, baseline PHQ-9, baseline GAD-7, waiting times referral to assessment, waiting time assessment to treatment as continuous covariates. Exact matching was used for all categorical covariates.

In the exploratory subgroup analyses, the interactions tests indicated no strong evidence that associations between socio-demographic factors and outcomes were modified by ADHD diagnosis status, although smaller sample sizes of older age groups and minority ethnic groups limit the ability to draw firm conclusions. For example, people from both male and female gender with ADHD had lower odds of achieving reliable improvement (Interaction Test p = 0.0954, AdjOR = 0.83 95% CI 0.78–0.88 p < 0.0001, Female: AdjOR = 0.78 95% CI 0.72–0.82 p < 0.0001 for females) reliable recovery (Interaction test p = 0.003, Male: AdjOR = 0.80 95% CI 0.75–0.85 p < 0.0001, Female: AdjOR = 0.70 95% CI 0.66–0.74 p < 0.0001), and higher odds of reliable deterioration (Interaction test p = 0.1420, Male: 1.26 95% CI 1.11–1.40 p < 0.0001, Female AdjOR = 1.43 95% CI 1.25–1.61 p < 0.0001) than the comparison group.

Sensitivity analyses including anxiety disorder specific measures and including a random intercept to account for NHS clinical entity yielded similar results (Appendix p20).

## Discussion

To our knowledge, this study is the first to evaluate the effectiveness of psychological therapies for depression and anxiety in a large cohort of adults with formally identified ADHD in a database of national coverage. We have found that adults with ADHD may experience a reduction in their symptoms of anxiety, depression, or both after a course of therapy. Yet there is an inequality in the likelihood of reaching favourable therapy outcomes and people with ADHD experienced poorer outcomes than those without ADHD, regardless of adjustment for covariates and propensity score matching. These findings are clinically important because most people with ADHD and co-occurring mental health difficulties will be seen in non-specialist psychological therapy services, and because psychological therapies tailored to ADHD that may alleviate depression and anxiety are not widely available. There were also notable differences in demographic characteristics between the ADHD and comparison groups, potentially reflecting, for example, known differences such as a higher diagnoses rates of ADHD in men and people from White ethnic backgrounds,^3^ and higher likelihood of unemployment in people with ADHD.^5^

Results also provide new evidence that directly targeting improvement in depression or anxiety symptoms, may be associated with improvements in terms of mental health for this population. This is important because RCTs have mostly evaluated the reduction of anxiety and depression through intervening in core ADHD traits, in study populations that may not experience high depression or anxiety. Our pre-post effect sizes were higher or comparable than such RCTs, although it is important to consider that because people in our study experienced depression and anxiety above clinical caseness thresholds, the increased magnitude of effect sizes in our findings could be partly explained by natural variations in symptoms scores (regression to the mean). Given the variety of both psychological (e.g., chronicity) factors and social (e.g., stigma and discrimination) factors affecting the experience of anxiety and depression in ADHD, this study suggests it can also be beneficial to treat anxiety and depression. Findings echo evidence from other neurodivergent populations, as research has recommended that autistic people are offered interventions directly addressing mental health difficulties while adopting person-centred care principles that account for the experience of being autistic.^35^

The finding that therapy outcomes are poorer for people with ADHD is also an important new finding. There are several potential explanations for the differences observed between the ADHD and comparison group. Firstly, because of the neurobiological nature of ADHD, it is possible that mechanisms of change in psychological therapy are different in people with ADHD, making it plausible that treatment protocols designed for the general population may not be effective without adaptation. There is also evidence that people with ADHD may be at risk of increased chronicity of mental health conditions, which could explain why interventions may be less effective.^5^ People with ADHD also cited the lack of awareness of ADHD as a barrier to a positive experience of therapy.^36^ This lack of awareness could lead some people with ADHD to feeling misunderstood or inadequate in therapy, or to struggle to apply therapy strategies in their daily life, which in some cases, may worsen difficulties with their mental health. It is also possible that without adaptations, ADHD traits may affect the experience of therapy, and people may have difficulties engaging with reading material, difficulties remembering to practice techniques in between sessions, or difficulties with sessions length or attendance, which all may alter engagement with therapy and could explain our finding of both earlier discharges from therapy and poorer therapy outcomes. A lack of standardized and evidence-based guidance for clinicians on adapting psychological therapies for people with ADHD means opportunities to benefit from therapy may have been limited.

This study used a database of national coverage, meaning study findings are generalisable to the population of people with ADHD who receive therapy to specifically address difficulties with depression and anxiety, provided their ADHD status is documented in their medical record. It is important to note that the prevalence of ADHD was lower in this study cohort than would be expected. This potential under-representation could reflect stigma and lack of knowledge as barriers to seeking support.^37^ Since ADHD in adulthood is underdiagnosed, this study cannot tell us how living with undiagnosed ADHD may affect therapy outcomes. Given underdiagnosis in older adults, women and people from ethnic minority groups,^3^ generalisability of findings to these groups may also be limited.

This study has several limitations. First, because of the observational nature of this research, this limited our capacity to handle bias. We used propensity-score matching to mitigate these limitations, caution should nonetheless be taken in the interpretation of as associations found here as they may not result from causal mechanisms. Exerting caution with regards to causality is especially important when considering the role of covariates that are modifiable by services in the associations, such as the number, type or frequency of sessions. In addition, NHS TTad represent the standard of care, and because data collected routinely may be associated with therapy outcomes, no negative control exposure or outcomes could be identified, which constitute further limits to causal interpretations of our findings. Secondly, the use of electronic healthcare records means that a substantial proportion of covariate data was missing, and limits the generalisability of the findings as outlined above. Thirdly, people with ADHD may take medication to manage difficulties associated with ADHD, but information on the type, dose, and other specific details pertaining to medication were not available in our study. Meta-analytic evidence of RCTs of CBT designed to help with ADHD management suggests that taking ADHD medication is associated with similar reductions in depression scores, but larger reductions in anxiety scores, compared to not taking ADHD medication.^33^ This warrants more research to understand whether and how taking ADHD medication may affect therapy outcomes for people experiencing clinical levels of depression or anxiety. Similarly, we do not know what kind of non-pharmacological support is available for people to manage ADHD, so it is unknown whether people in our cohort may have been familiar with interventions such as CBT for ADHD. It is also unknown whether alleviating depression and anxiety may also support people in terms of ADHD management. Finally, we had no information about the specificity of ADHD presentations, so we cannot tell whether specific features of ADHD in terms of attention, impulsivity or hyperactivity may be associated with different therapy outcomes.

### Conclusion

Together, these findings suggest the importance of building a better evidence base for mental health interventions in people ADHD with co-occurring depression and anxiety. Therapy modules could be developed to consider the specific interactions between ADHD and depression or anxiety. Findings suggest that such interventions may be provided in non-specialist mental health services but highlight a need for evidence-based guidance for clinicians to adapt therapy for people with ADHD which could consider both strengths and difficulties associated with living ADHD. Such adaptations could improve both therapeutic relationships and retention in the interventions. Given that some groups face both systemic barriers to accessing ADHD diagnosis and mental health care understanding psychological impact of systemic stressors faced by people with ADHD across the lifespan may also be also important to consider to design more representative, and effective interventions and provide more equitable care.

## Contributors

CEB, JSW, RS, JS, and AJ were involved in the conceptualisation and design of the study. CEB, JWS, AJ, JS contributed to the data curation. CEB did the formal analysis, visualisations, and wrote the original draft. AJ and JS contributed to funding acquisition. CEB, AJ, JS, JSW, RS and JB contributed to the methodology. CEB did the project administration, AJ and JS contributed to supervision. CEB, AJ, JSW and JS had full access to and verified the data. CEB wrote the original draft of the manuscript. All authors were involved in the review and editing of subsequent version of the manuscript. All authors accept the responsibility to submit for publication.

## Data sharing statement

All data used for this study are available upon successful application to NHS Digital via the Data Access Request Service (DARS): https://digital.nhs.uk/services/data-access-request-service-dars. Click or tap if you trust this link. Data fields can be accessed via NHS Digital data dictionary: https://www.datadictionary.nhs.uk/. Click or tap if you trust this link.

## Declaration of interests

J.E.J.B received research funding from the Royal College of Psychiatrists, the NIHR (National Institute of Health Research), and the Medical Research Council (MRC), all unrelated to this work.

J.E.J.B also received payment or honoraria from the Catholic University of Louvain and the University of Valencia, all unrelated to this work.

H.C declares no conflict of interest.

D.D received research funding from the NIHR unrelated to this work.

W.D declares no conflict of interest.

C.E.B received research funding from the NIHR unrelated to this work.

N.H received a salary from Everyturn and Vita Health Group for unrelated work. N.H is also in private practice through CBT for ADHD which is unrelated to this work. N.H received payment or honoraria from the University of Surrey, the BABCP, SBK, BESPOKE CBT Low intensity conference, South London and Maudsley Talking Therapies, West London NHS Talking Therapies, all unrelated to this work. N.H is involved in a voluntary capacity with the BABCP, the Kent and Medway neurodivergence pathway group, and NHS England.

A.J received research funding from the NIHR unrelated to this work.

W.M received funding from the Economic and Social Research Council (ESRC), the NIHR and Dunhill Medical Trust, all unrelated to this work.

D.O declares no conflict of interest.

A.M.R.P received research funding from the NIHR unrelated to this work.

G.P received research funding from the NHS and UKRI, unrelated to this work.

L.P received research funding from the Australian Research Council, the Simons Foundation, the NIHR and the National Health and Medical Research Council (NHMRC), all unrelated to this work. L.P also received financial support from the Autism Europe Congress, unrelated to this work. L.P is the research chair of the Pathological Demand Avoidance Society, unrelated to this work.

S.P declares no conflict of interest.

A.R received research funding from the NIHR unrelated to this work.

R.S received research funding from the Royal College of Psychiatrists, the NIHR and holds an honorary senior information analyst position with NHS England, all unrelated for this work.

H.S declares no conflict of interest.

G.W received research funding from the British Academy, unrelated to this work.

J.S received research funding from the NIHR, unrelated to this work.

J.W.S declares no conflict of interest.
