# Supplementary material for: Effectiveness of psychological therapies for depression and anxiety in adults with and without ADHD in England: a retrospective cohort study
Source: Lancet Reg Health Eur. 2026 Aug 6;68:101798. doi: 10.1016/j.lanepe.2026.101798 (PMC13475375; doi:10.1016/j.lanepe.2026.101798)
Supplement: Supplementary file 1 [file mmc1.docx]

**APPENDIX**

[Appendix A	MODIFY Study Dataset	2](#_Toc233878215)

[Appendix B	NHS Talking Therapies for Anxiety and Depression Services	3](#_Toc233878216)

[Appendix C	Covariates used in analyses	5](#_Toc233878217)

[Appendix D	Matching Procedure	7](#_Toc233878218)

[Appendix E	Effect sizes from studies of Cognitive-Behavioural Therapy conducted in people with ADHD, including anxiety or depression as secondary outcomes	9](#_Toc233878219)

[Appendix F	Subgroup analyses	12](#_Toc233878220)

[Appendix G	Sensitivity analysis: using anxiety disorder specific measures when available instead of the GAD	18](#_Toc233878221)

[Appendix H	Sensitivity Analysis – Defining the ADHD Group as people with the first occurrence of a diagnosis code of ADHD before NHS TTad	20](#_Toc233878222)

[Appendix I	Sensitivity analysis: additionally adjusting for clinical commissioning group (NHS service)	21](#_Toc233878223)

[Appendix J	The RECORD statement – checklist of items, applied to the present study	22](#_Toc233878224)

# Appendix A MODIFY Study Dataset

| **IAPT dataset ^1^** | Routinely collected data for every patient seen in NHS TTad (formerly called IAPT) services across all England between the 1^st^ April 2012 and the 31^st^ March 2022. Includes demographic (e.g., gender, age, ethnicity), therapy (e.g., referral and assessment dates, treatment information at each appointment) and outcome (improvement, recovery, deterioration) information for individual patients. |
| --- | --- |
| **Hospital Episode Statistics dataset ^2^** | Admitted Patient Car and Outpatient datasets from all National Health Service (NHS) hospitals across England. Includes demographic (e.g., age, ethnicity, gender), geographical (e.g., residential area, area treatment received), administrative (e.g., dates of admission and discharge), and clinical (e.g., diagnoses, treatments, operations) information for individual patients.  The Accident and Emergency and Emergency Care Data Set were also used for linkage purposes only. |
| **Mental Health Services dataset ^3^** | Previously known as the Mental Health Minimum Dataset (MHMDS) and the Mental Health and Learning Disability Dataset (MHLDDS). Includes data from secondary care services (e.g., provided in hospitals, outpatient clinics, in the community) for mental illness, learning disability, autism, and other neurodevelopmental conditions. |
| **HES-ONS Mortality dataset ^4^** | Linked information from HES and Office of National Statistics (ONS) mortality data. Includes cause, date, and place of death (both in and out of hospital). |

# Appendix B NHS Talking Therapies for Anxiety and Depression Services

After an initial assessment, and prior to treatment start, the clinician and the person receiving treatment agree on which condition therapy will focus on. Many people will meet diagnostic criteria for several mental health conditions, and our dataset captures information on the condition being treated. People with less complex problems are commonly offered short-term (<=8 sessions) “low intensity” guided self-help therapies, which can be delivered face to face or remotely, in one-to-one or group settings. Where people do not respond to low intensity therapy or their problems are more complex at referral, individuals are commonly offered high intensity therapies (>=16-20 sessions), which are formulation driven one-to-one cognitive behavioural or other types of evidence-based therapies. Interventions are standardized and delivered by trained practitioners and psychotherapists following evidence-based protocols ^5^.

|  | **Condition** | **Psychological therapies** | **Source** |
| --- | --- | --- | --- |
| Step 2: Low-intensity interventions | Depression | Individual guided self-help based on cognitive behavioural therapy (CBT), computerised CBT, behavioural activation, structured group physical activity programme | NICE guidelines NG222, CG91 |
|  | Generalised anxiety disorder | Self-help, or guided self-help based on CBT, psycho-educational groups, computerised CBT | NICE guidelines CG113 |
|  | Panic disorder | Self-help, or guided self-help based on CBT, psycho-educational groups, computerised CBT | NICE guidelines CG113 |
|  | Low impact Obsessive-compulsive disorder | Guided self-help based on CBT | NICE guidelines CG31 |
| Step 3: High intensity interventions | Depression, Chronic depression, prevention of relapse  For individuals with mild to moderate severity who have not responded to initial low-intensity interventions | CBT (individual or group) or interpersonal therapy (IPT)  Behavioural Activation  Couple therapy  Counselling for depression  Brief psychodynamic therapy  Note: Psychological interventions can be provided in combination with antidepressant medication | NICE guidelines: NG222, CG91 |
|  | Depression  Moderate to severe | CBT (individual) or IPT, each with medication | NICE guidelines: NG222, CG91 |
|  | Generalised anxiety disorder | CBT applied relatasion | NICE Guidelines: CG113 |
|  | Social Anxiety Disorder | CBT (individual) for social anxiety disorder | NICE guidelines: CG159 |
|  | Post-traumatic stress disorder | Trauma focussed CBT (Individual)  EMD (in specific situations) | NICE guidelines: NG116 |
|  | Health anxiety | CBT | No NICE guideline |

Source: National Collaborating Centre for Mental Health. The Improving Access to Psychological Therapies Manual 2024.

# Appendix C Covariates used in analyses

A range of covariates known to be associated with therapy outcomes^6-10^ were included in analyses. Age was fitted as a categorical variable in the model to account for its non-linear association with therapy outcomes.

| **Variables** | **Identification in databases** | Data source |  | | **Inclusion as covariate in analyses** | | | | | | | |
| --- | --- | --- | --- | --- | --- | --- | --- | --- | --- | --- | --- | --- |
|  |  |  | Model # | | | | | | | | PS estimation model | |
|  |  |  | 1 | 2 | | | 3 | | 4 | |  | |
|  |  |  | Full cohort | Full cohort | | | Full cohort | | PS-Matched cohort | |  |  |
| **Exposure** | | | | | | | | | | | | |
| ADHD diagnosis code | Identified using diagnostic codes according to the International Classification of Diseases, 10th Revision (ICD-10) using codes F90.0, F90.1, F90.2, F90.8, F90.9 | HES APC/HES OP/ MHSDS | x |  | | |  | |  | | N/A | |
| **Covariates** | | | | | | | | | | | | |
| Demographic covariates | Self-reported measures collected at the point of referral: gender, age, index of multiple deprivation (IMD) quintile (a lowest IMD indicates a higher deprivation area), ethnicity (based on UK census codes ‘White’, ‘Mixed’, ‘Asian’, ‘Black’, ‘Chinese’ and ‘other’), and employment status ( employed, unemployed and seeking, unemployed and not seeking work, unemployed, long-term sickness or disability) were available in the dataset. | NHS TTad |  | x | | | x | | x | | x | |
| Long-term health conditions | All patients are asked whether they have any long-term physical health condition (LTC) at referral. The type of condition was not available in the dataset. Presence of a long-term condition may be associated with an adaptation in the therapy provided. | NHS TTad |  | x | | | x | | x | | x | |
| Psychotropic Medication taken at start of treatment | Clinicians in the services routinely record whether their patients were prescribed psychotropic medication(s) before treatment. | NHS TTad |  | x | | | x | | x | | x | |
| **Treatment factors** | |  |  |  | |  | |  | |  | |  |
| NHS TTad treatment factors | The number of treatment sessions received, Reason for treatment discontinuation (Completed, Deceased, Declined, Dropout, Mutual agreement or signposting, Service not suitable, Referred on), Year of referral, Number of high intensity treatment session, number of low intensity treatment sessions, frequency of sessions / week | NHS TTad |  |  | | | x | | x | | x | |

Abbreviations: HES=Hospital Episode Statistics, IAPT = Improving Access to Psychological Therapies, ICD = International Classification of Disease, MHSDS=Mental Health Services Dataset, N/A = Not applicable, PS = Propensity Score

# Appendix D Matching Procedure

In the presence of a vast pool of potential controls available (~3 million), efforts were made to seek exact matching when possible for categorical covariates, to achieve balance across characteristics also investigated in subgroup analyses. A propensity score was used to find the most appropriate control based on the remaining covariates where exact matching was not used. The propensity score was estimated using logistic regression by modelling the probability to belong to the ADHD group (Table D1). The model included all factors thought to be associated with the outcomes.

The matching algorithm occurred in two steps. First, exact matching was carried out based on the following categorical covariates: age group, ethnicity, employment status, IMD quintile, taking psychotropic medication at referral, reporting a long-term health condition. This led to the stratification of the sample in 2634 clusters, based on unique combinations of the exact matching covariates. Propensity score matching was then performed in each cluster, allowing for replacement and using a caliper. The quality of the matching was assessed using balancing checks and by comparing demographic characteristics of the ADHD group before and after matching.

This algorithm was repeated using different combinations of exact matching covariates, and by varying the value of the caliper (from 0·001 to 0·2), and investigated the possibility of both 1:1 and 1:2 matching. The final matched cohort was chosen on the basis of maximising the number of individuals in the ADHD group, obtaining a matched comparison individual, whilst minimising differences in baseline characteristics between individuals with ADHD and their controls. Matching was done without replacement, a robust variance estimator was used in regressions.

**Table D1: Logistic regression model – probability to belong to the ADHD group**

|  | **Coefficient** | **Standard error** | **p-value** |
| --- | --- | --- | --- |
| Age Group (18-24 = ref) |  |  |  |
| 25-44 | -0.83 | 0.030 | <.0001 |
| 45-64 | -2.38 | 0.036 | <.0001 |
| 65+ | -3.66 | 0.082 | <.0001 |
| Ethnicity (ref=white) |  |  |  |
| Mixed | 0.11 | 0.093 | <.0001 |
| Asian | -1.16 | 0.070 | <.0001 |
| Black | -0.69 | 0.081 | <.0001 |
| Missing | -0.38 | 0.043 | 0.0001 |
| IMD quintile (ref=1^st^) |  |  |  |
| 2 | -0.06 | 0.032 | <.0001 |
| 3 | -0.13 | 0.037 | <.0001 |
| 4 | -0.14 | 0.041 | <.0001 |
| 5 | -0.09 | 0.049 | <.0001 |
| Missing | -0.11 | 0.069 | <.0001 |
| Employment status (ref=employed) |  |  |  |
| Unemployed, seeking work | 0.73 | 0.039 | <.0001 |
| Unemployed, not seeking work | 0.35 | 0.041 | <.0001 |
| Long-term illness/benefits | 0.82 | 0.040 | <.0001 |
| Missing | 0.22 | 0.057 | <.0001 |
| Gender (ref male) |  |  |  |
| Female | -0.77 | 0.025 | 0.0307 |
| Presenting problem (ref=Anxiety) |  |  |  |
| Depression | ref |  |  |
| Mixed Anxiety and Depression | -0.05 | 0.029 | 0.1164 |
| GAD | -0.001 | 0.027 | 0.9626 |
| OCD | 0.12 | 0.070 | 0.0903 |
| PTSD | 0.047 | 0.050 | 0.3488 |
| Phobic anxiety and panic | -0.25 | 0.042 | <.0001 |
| Unspecified anxiety | -0.13 | 0.084 | 0.1286 |
| Missing | 0.18 | 0.025 | <0.0001 |
| Taking psychotropic medication at referral (ref=No) |  |  |  |
| Yes | 0.22 | 0.019 | <0.001 |
| Missing | 0.10 | 0.032 | 0.0610 |
| Self-reported long-term health condition (ref=No) |  |  |  |
| Yes | 0.38 | 0.022 | <0.001 |
| Missing | 0.04 | 0.022 | 0.0016 |
| Time between referral and assessment | 0.17 | 0.001 | <0.0001 |
| Time between assessment and treatment | 0.007 | 0.0007 | <0.0001 |
| Appointment year | 0.002 | 0.004 | 0.5142 |
| Number of high intensity sessions | 0.010 | 0.002 | <0.0001 |
| Number of low intensity sessions | -0.29 | 0.004 | <0.0001 |
| Frequency of sessions (/week) | 0.015 | 0.004 | 0.004 |
| Reason for discharge |  |  |  |
| Deceased | 0.036 | 0.220 | 0.8710 |
| Mutual agreement | 0.204 | 0.068 | 0.0027 |
| Service not suitable / referred on | 0.66 | 0.032 | <0.0001 |
| Unscheduled discontinuation | 0.18 | 0.022 | <0.0001 |
| Missing | 0.24 | 0.026 | <0.0001 |
| Depression score at baseline (PHQ-9) | 0.027 | 0.002 | <0.0001 |
| Anxiety score at baseline (GAD-7) | 0.023 | 0.003 | <0.0001 |
| Intercept | -10.36 | 7.32 | 0.1570 |

# Appendix E Effect sizes from studies of Cognitive-Behavioural Therapy conducted in people with ADHD, including anxiety or depression as secondary outcomes

|  |  | **CBT in people with ADHD - pre and post mean (SD)** | | | | | **Cohens'dav (calculated by us)** |
| --- | --- | --- | --- | --- | --- | --- | --- |
| **Study** | **Measure used for depression or anxiety** | **M (Pre)** | **SD (Pre)** | **M (Post)** | **SD (Post)** | **N** |  |
| **Depression** | | | | | | | |
| Dittner 2018 | Hospital Anxiety and Depression Scale - Depression | 5.5 | 3 | 3.1 | 2.3 | 30 | -0.83 (-1.36 to -0.3) |
| Emilsson 2011 | Beck Depression inventory | 11.6 | 8.71 | 7.22 | 6.84 | 27 | -0.52 (-1.06 to 0.02) |
| Fleming 2015 | Beck Depression inventory | 10.65 | 7.86 | 7.47 | 5.94 | 17 | -0.42 (-1.1 to 0.26) |
| Gu 2018 | Beck Depression inventory | 9.79 | 4.57 | 7.07 | 2.71 | 28 | -0.65 (-1.19 to -0.11) |
| Hepark 2019 | Beck Depression inventory | 12.1 | 8.7 | 9.1 | 7.8 | 55 | -0.34 (-0.72 to 0.04) |
| Halmoy 2022 | Beck Depression inventory | 20.9 | 11.2 | 14.5 | 11.8 | 50 | -0.52 (-0.92 to -0.12) |
| Huang 2019 (CBT) | Self-rating depression scale | 40.98 | 8.29 | 36.75 | 10.69 | 43 | -0.41 (-0.84 to 0.02) |
| Huang 2019 (CBT Booster) | Self-rating depression scale | 41.26 | 10.09 | 34.07 | 9.96 | 43 | -0.67 (-1.11 to -0.24) |
| Jang 2021 | Quick Inventory of Depressive Symptomatology | 13.91 | 7.31 | 10.63 | 7.18 | 23 | -0.42 (-1.01 to 0.16) |
| Moëll 2015 | Hospital Anxiety and Depression Scale - Depression | 7.08 | 4.21 | 6.62 | 4.54 | 29 | -0.1 (-0.61 to 0.42) |
| Nasri 2023 | Montgomery-Åsberg Depression Rating Scale | 16.4 | 8.3 | 11.3 | 7.81 | 36 | -0.59 (-1.06 to -0.12) |
| Pan 2021 | Self-rating depression scale | 41.71 | 9.39 | 36.86 | 8.78 | 49 | -0.5 (-0.9 to -0.1) |
| Pettersson 2017 (iCBT Group) | Beck Depression inventory | 18.64 | 13.44 | 11.97 | 9.4 | 14 | -0.53 (-1.28 to 0.23) |
| Pettersson 2017 (iCBT Individual) | Beck Depression inventory | 17.85 | 14.87 | 14.01 | 15.73 | 13 | -0.23 (-1.01 to 0.54) |
| Philipsen 2015 (DBT) | Beck Depression inventory | 12.3 | 8.8 | 9.9 | 8.4 | 109 | -0.26 (-0.53 to 0.01) |
| Philipsen 2015 (DBT + Med) | Beck Depression inventory | 11.8 | 8.4 | 9.8 | 8.5 | 107 | -0.22 (-0.49 to 0.05) |
| Safren 2005 | Beck Depression inventory | 11.88 | 8.07 | 7.63 | 4.26 | 16 | -0.58 (-1.29 to 0.13) |
| Schönenberg 2017 | Beck Depression inventory | 20.71 | 14.2 | 11.14 | 8.9 | 38 | -0.73 (-1.19 to -0.26) |
| Solanto 2010 | Beck Depression inventory | 11.48 | 9.59 | 9.66 | 8.31 | 45 | -0.19 (-0.6 to 0.22) |
| van der Oord 2020 | Beck Depression inventory | 9.33 | 8.91 | 6.14 | 6.39 | 30 | -0.38 (-0.89 to 0.13) |
| van Emmerik-van Oortmerssen 2019 | Beck Depression inventory | 15.05 | 12.55 | 8.39 | 10.56 | 60 | -0.53 (-0.9 to -0.17) |
| Vidal 2013 | Beck Depression inventory | 12.13 | 9.34 | 12.4 | 11.07 | 15 | 0.02 (-0.69 to 0.74) |
| Virta 2010 | Beck Depression inventory | 13.6 | 5.6 | 9 | 8.3 | 10 | -0.59 (-1.49 to 0.3) |
| Young 2015 | Beck Depression inventory | 12.72 | 8.62 | 8.38 | 6.99 | 48 | -0.51 (-0.92 to -0.11) |
| Tsirmpas 2023 | PHQ-9 | 14.3 | 4.22 | 7.7 | 5.26 | 30 | -1.28 (-1.84 to -0.73) |
| The present study | PHQ-9 | 17.37 | 5.15 | 12.58 | 6.95 | 13693 | -0.72 (-0.75 to -0.7) |
| **Anxiety** | | | | | | | |
| Dittner 2018 | Hospital Anxiety and Depression Scale - Anxiety | 10.3 | 3.5 | 7.5 | 3.5 | 30 | -0.73 (-1.25 to -0.21) |
| Emilsson 2011 | Beck Anxiety Inventory | 13.43 | 8.67 | 11 | 10.61 | 27 | -0.23 (-0.76 to 0.31) |
| Fleming 2015 | Beck Anxiety Inventory | 14.18 | 13.69 | 9.82 | 9.25 | 17 | -0.33 (-1.01 to 0.34) |
| Gu 2018 | Beck Anxiety Inventory | 14 | 3.65 | 9.5 | 6.05 | 28 | -0.79 (-1.34 to -0.25) |
| Halmoy 2022 | Beck Anxiety Inventory | 15.5 | 9.7 | 13.4 | 11.2 | 50 | -0.19 (-0.58 to 0.21) |
| Huang 2019 (CBT) | Self-rating anxiety scale | 37.39 | 7.45 | 31.52 | 7.51 | 43 | -0.72 (-1.15 to -0.28) |
| Huang 2019 (CBT Booster) | Self-rating anxiety scale | 38.64 | 8.98 | 31.38 | 10.31 | 43 | -0.68 (-1.12 to -0.25) |
| Jang 2021 | Self-rating anxiety scale | 38.09 | 10.29 | 34.42 | 9.51 | 23 | -0.34 (-0.92 to 0.24) |
| Moëll 2015 | Hospital Anxiety and Depression Scale - Anxiety | 8.85 | 3.95 | 7.96 | 4.1 | 29 | -0.2 (-0.72 to 0.31) |
| Pan 2021 | Self-rating anxiety scale | 36.22 | 8.33 | 31.82 | 8.06 | 49 | -0.49 (-0.89 to -0.09) |
| Pettersson 2017 (iCBT Group) | Beck anxiety inventory | 12.43 | 5.94 | 15.2 | 8.04 | 14 | 0.35 (-0.39 to 1.1) |
| Pettersson 2017 (iCBT Individual) | Beck anxiety inventory | 17.31 | 15.64 | 13.79 | 14.59 | 13 | -0.21 (-0.98 to 0.56) |
| Safren 2005 | Beck anxiety inventory | 6.19 | 7.17 | 3.69 | 3.52 | 16 | -0.38 (-1.08 to 0.32) |
| Solanto 2010 | Hamilton anxiety rating scale | 9.56 | 5.37 | 8.07 | 5.38 | 45 | -0.25 (-0.67 to 0.16) |
| van Emmerik-van Oortmerssen 2019 | Beck Anxiety Inventory | 15.83 | 14.79 | 10.07 | 12.02 | 60 | -0.39 (-0.75 to -0.03) |
| Young 2015 | Beck Anxiety Inventory | 13.84 | 8.69 | 10.41 | 9.1 | 48 | -0.35 (-0.75 to 0.05) |
| Tsirmpas 2023 | GAD-7 | 11.4 | 4.76 | 6.1 | 4.55 | 30 | -1.04 (-1.58 to -0.5) |
| The present study | GAD-7 | 15.26 | 4.3 | 11.07 | 6.04 | 13693 | -0.72 (-0.74 to -0.69) |
| Descriptive results extracted from meta-analyses:  Young Z, Moghaddam N, Tickle A. The Efficacy of Cognitive Behavioral Therapy for Adults With ADHD: A Systematic Review and Meta-Analysis of Randomized Controlled Trials. Journal of Attention Disorders. 2020;24(6):875–88.  Liu Y, Zhu F, Yu Y, Song Y, Luo H, Xu B, et al. A meta-analysis of the intervention effect of cognitive behavioral therapy on adult ADHD. Journal of Affective Disorders. 2026;399:121107.  Cohen'd calculated by us. | | | | | | | |
| Case definitions: HADS: 11 or higher, Beck's depression: >=14, Beck's anxiety: >=16, SDS: 50 or higher, SAS: 50 or higher | | | | | | | |

# Appendix F Subgroup analyses

A – Adjusted reliable improvement, recovery and deterioration rates by subgroup - PS-matched cohort
 **Reliable improvement rates (%)	Reliable recovery rates (%)	Reliable deterioration rates (%)**

**Reliable improvement rates (%)	Reliable recovery rates (%)	Reliable deterioration rates (%)**

**Reliable improvement rates (%)	Reliable recovery rates (%)	Reliable deterioration rates (%)**

Abbreviations: IMD = Index of multiple deprivation, M.A.D: Mixed anxiety and depression, GAD = Generalised anxiety disorder, PTSD = post-traumatic stress disorder, Unspec Anx=unspecified anxiety, LTC=Long-term condition

B – Adjusted reliable improvement, recovery and deterioration rates by subgroup – Full cohort
**Reliable improvement rates (%)	Reliable recovery rates (%)	Reliable deterioration rates (%)**

**Reliable improvement rates (%)	Reliable recovery rates (%)	Reliable deterioration rates (%)**

**Reliable improvement rates (%)	Reliable recovery rates (%)	Reliable deterioration rates (%)**

Abbreviations: IMD = Index of multiple deprivation, M.A.D: Mixed anxiety and depression, GAD = Generalised anxiety disorder, PTSD = post-traumatic stress disorder, Unspec Anx=unspecified anxiety, LTC=Long-term condition

# Appendix G Sensitivity analysis: using anxiety disorder specific measures when available instead of the GAD

**Anxiety disorder specific measures and caseness cut-offs**

| **Symptom Scale** | **Caseness** | **Change (improvement/deterioration)** |
| --- | --- | --- |
| Patient Health Questionnaire | ≥10 | 6 |
| Generalised Anxiety Disorder Scale | ≥8 | 4 |
| Agoraphobia Morbidity Inventory | ≥2.3 | 0.73 |
| Social Phobia Inventory | ≥19 | 10 |
| Panic Disorder Severity Scale | ≥8 | >5 |
| Impact of Events Scale (PTSD) | 33 | 9 |
| Obsessive Compulsive Inventory | ≥40 | 32 |
| Health Anxiety Inventory | ≥18 | 4 |

**Outcomes:**

| **Total N=3 422 396** | **Reliable improvement** | | | | **Reliable recovery** | | | | | **Reliable Deterioration** | | | | |
| --- | --- | --- | --- | --- | --- | --- | --- | --- | --- | --- | --- | --- | --- | --- |
|  | N Events / N in Group (% with event) | | | | N Events / N in Group (% with event) | | | | N Events / N in Group (% with event) | | | | |  |
| **ADHD Group** | 8 287 / 14 329 (57.8%) | | | | 4 212 / 14 329 (29.4%) | | | | 1 281 / 14 329 (8.9%) | | | | |  |
| **No Identified ADHD** | 2 384 938 / 3 408 067 (70.0%) | | | | 1 616 067 / 3 408 067 (47.4%) | | | | 194 316 / 3 408 067 (5.7%) | | | | |  |
| **Models** | N | OR | 95% CI | p-value | N | OR | 95% CI | p-value | N | | OR | 95% CI | p-value |  |
| **Model 1 - Full sample (unadjusted)** | 3 422 396 | 0.59 | 0·57 **–** 0·61 | <·0001 | 3 422 396 | 0.46 | 0.45 – 0.48 | <.0001 | 3 422 396 | | 1.62 | 1.53 – 1.72 | <.0001 |  |
| **Model 2 - Full sample (adjusted)^a^** | 3 422 396 | 0.73 | 0.70 – 0.75 | <.0001 | 3 422 396 | 0.65 | 0.63 – 0.68 | <.0001 | 3 422 396 | | 1.46 | 1.38 – 1.55 | <.0001 |  |
| **Model 3 - Full sample (adjusted)^b^** | 3 422 396 | 0.80 | 0.77 – 0.83 | <.0001 | 3 422 396 | 0.71 | 0.68 – 0.74 | <.0001 | 3 422 396 | | 1.32 | 1.24– 1.40 | <.0001 |  |
| Abbreviations: OR=Odds ratio, CI=Confidence Interval, PHQ=Patient Health Questionnaire, GAD=General Anxiety Disorder, PS = Propensity Score  Missing data for categorical covariates was imputed by using a “missing” category· There was no missing data for continuous covariates.  ^a^ Adjusted for gender, ethnicity, employment status, LTC case, psychotropic medication, diagnosis category, IMD quintile, year of referral, age at referral, baseline PHQ-9,  ^b^ Adjusted for gender, ethnicity, employment status, LTC case, psychotropic medication, diagnosis category, IMD quintile, year of referral, age at referral, baseline PHQ-9, waiting times referral to assessment, waiting time assessment, year of appointment, number of low intensity sessions, number of high intensity sessions, reason for ending treatment, frequency of sessions | | | | | | | | | | | | | |  |

# Appendix H Sensitivity Analysis – Defining the ADHD Group as people with the first occurrence of a diagnosis code of ADHD before NHS TTad

| **Total N= 3 257 017** | **Reliable improvement** | | | | **Reliable recovery** | | | | | **Reliable Deterioration** | | | | |
| --- | --- | --- | --- | --- | --- | --- | --- | --- | --- | --- | --- | --- | --- | --- |
|  | N Events / N in Group (% with event) | | | | N Events / N in Group (% with event) | | | | N Events / N in Group (% with event) | | | | |  |
| **ADHD Group** | 3 154 / 5 418 (58.2%) | | | | 1 644 / 5 418 (30.3%) | | | | 493 / 5 418 (9.10%) | | | | |  |
| **No Identified ADHD** | 2 226 524 / 3 251 599 (69.7%) | | | | 1 541 252 / 3 251 599 (47.4%) | | | | 187 871 / 3 251 599 (5.78%) | | | | |  |
|  | N | OR | 95% CI | p-value | N | OR | 95% CI | p-value | N | | OR | 95% CI | p-value |  |
| **Model 1 - Full sample (unadjusted)** | 3 257 017 | 0.61 | 0.57 – 0.64 | <·0001 | 3 257 017 | 0.48 | 0.46 – 0.51 | <.0001 | 3 257 017 | | 1.63 | 1.48-1.79 | <.0001 |  |
| **Model 2 - Full sample (adjusted)^a^** | 3 257 017 | 0.77 | 0.73 – 0.82 | <.0001 | 3 257 017 | 0.73 | 0.69 -0.77 | <.0001 | 3 257 017 | | 1.47 | 1.33-1.60 | <.0001 |  |
| **Model 3 - Full sample (adjusted)^b^** | 3 257 017 | 0.83 | 0.77 – 0.87 | <.0001 | 3 257 017 | 0.77 | 0.72 – 0.82 | <.0001 | 3 257 017 | | 1.36 | 1.24-1.50 | <.0001 |  |
| Abbreviations: OR=Odds ratio, CI=Confidence Interval, PHQ=Patient Health Questionnaire, GAD=General Anxiety Disorder  Missing data for categorical covariates was imputed by using a “missing” category· There was no missing data for continuous covariates.  ^a^ Adjusted for gender, ethnicity, employment status, LTC case, psychotropic medication, diagnosis category, IMD quintile, year of referral, age at referral, baseline PHQ-9,  ^b^ Adjusted for gender, ethnicity, employment status, LTC case, psychotropic medication, diagnosis category, IMD quintile, year of referral, age at referral, baseline PHQ-9, waiting times referral to assessment, waiting time assessment, year of appointment, number of low intensity sessions, number of high intensity sessions, reason for ending treatment, frequency of sessions  ^c^ The propensity score was estimated using logistic regression using age group, gender, ethnicity, employment status, LTC case, psychotropic medication, IMD quintile as categorical covariates and year of first appointment, baseline PHQ-9, baseline GAD-7, waiting times referral to assessment, waiting time assessment to treatment as continuous covariates. Exact matching was used for all categorical covariates. | | | | | | | | | | | | | |  |

# Appendix I Sensitivity analysis: additionally adjusting for clinical commissioning group (NHS service)

|  | **Reliable improvement** | | | | **Reliable recovery** | | | | | **Reliable Deterioration** | | | | |
| --- | --- | --- | --- | --- | --- | --- | --- | --- | --- | --- | --- | --- | --- | --- |
|  | N | OR | 95% CI | p-value | N | OR | 95% CI | p-value | N | | OR | 95% CI | p-value |  |
| **Model 1 - Full sample (unadjusted)** | 3 257 017 | 0.59 | 0.57-0.61 | <.0001 | 3 257 017 | 0.46 | 0.44-0.48 | <.0001 | 3 257 017 | | 1.87 | 1.73-2.03 | <.0001 |  |
| **Model 2 - Full sample (adjusted)^a^** | 3 257 017 | 0.71 | 0.68-0.73 | <.0001 | 3 257 017 | 0.66 | 0.64-0.69 | <.0001 | 3 257 017 | | 1.32 | 1.22-1.43 | <.0001 |  |
| **Model 3 - Full sample (adjusted)^b^** | 3 257 017 | 0.78 | 0.75-0.81 | <0.001 | 3 257 017 | 0.72 | 0.69-0.75 | <.0001 | 3 257 017 | | 1.62 | 1.53-1.72 | <.0001 |  |
| Abbreviations: OR=Odds ratio, CI=Confidence Interval, PHQ=Patient Health Questionnaire, GAD=General Anxiety Disorder  Missing data for categorical covariates was imputed by using a “missing” category· There was no missing data for continuous covariates.  ^a^ Adjusted for gender, ethnicity, employment status, LTC case, psychotropic medication, diagnosis category, IMD quintile, year of referral, age at referral, baseline PHQ-9,  ^b^ Adjusted for gender, ethnicity, employment status, LTC case, psychotropic medication, diagnosis category, IMD quintile, year of referral, age at referral, baseline PHQ-9, waiting times referral to assessment, waiting time assessment, year of appointment, number of low intensity sessions, number of high intensity sessions, reason for ending treatment, frequency of sessions  ^c^ The propensity score was estimated using logistic regression using age group, gender, ethnicity, employment status, LTC case, psychotropic medication, IMD quintile as categorical covariates and year of first appointment, baseline PHQ-9, baseline GAD-7, waiting times referral to assessment, waiting time assessment to treatment as continuous covariates. Exact matching was used for all categorical covariates. | | | | | | | | | | | | | |  |

# Appendix J  The RECORD statement – checklist of items, applied to the present study

|  | **Item No.** | **STROBE items** | **Location in manuscript where items are reported** | **RECORD items** | **Location in manuscript where items are reported** |  |
| --- | --- | --- | --- | --- | --- | --- |
| **Title and abstract** | | | | | | |
|  | 1 | (a) Indicate the study’s design with a commonly used term in the title or the abstract (b) Provide in the abstract an informative and balanced summary of what was done and what was found | Abstract | RECORD 1.1: The type of data used should be specified in the title or abstract. When possible, the name of the databases used should be included.  RECORD 1.2: If applicable, the geographic region and timeframe within which the study took place should be reported in the title or abstract.  RECORD 1.3: If linkage between databases was conducted for the study, this should be clearly stated in the title or abstract. | Abstract, methods section  Title, Abstract  Abstract |  |
| **Introduction** | | | | | | |
| Background rationale | 2 | Explain the scientific background and rationale for the investigation being reported | Introduction |  | Introduction |  |
| Objectives | 3 | State specific objectives, including any prespecified hypotheses | Research questions 1, 2, 3 |  | Research questions 1, 2, 3 |  |
| **Methods** | | | | | | |
| Study Design | 4 | Present key elements of study design early in the paper | Methods section |  | Methods section |  |
| Setting | 5 | Describe the setting, locations, and relevant dates, including periods of recruitment, exposure, follow-up, and data collection | Methods section, Appendix |  | Methods section, Appendix |  |
| Participants | 6 | *(a) Case-control study* - Give the eligibility criteria, and the sources and methods of case ascertainment and control selection. Give the rationale for the choice of cases and controls  *(b) Case-control study* - For matched studies, give matching criteria and the number of controls per case | Methods / Study cohort, Exposure, Study flow chart, Procedures Appendix  Methods/Statistical Analysis, Appendix | RECORD 6.1: The methods of study population selection (such as codes or algorithms used to identify subjects) should be listed in detail. If this is not possible, an explanation should be provided.  RECORD 6.2: Any validation studies of the codes or algorithms used to select the population should be referenced. If validation was conducted for this study and not published elsewhere, detailed methods and results should be provided.  RECORD 6.3: If the study involved linkage of databases, consider use of a flow diagram or other graphical display to demonstrate the data linkage process, including the number of individuals with linked data at each stage. | Methods / Study Cohort/Exposure  Methods  Study flow chart Methods |  |
| Variables | 7 | Clearly define all outcomes, exposures, predictors, potential confounders, and effect modifiers. Give diagnostic criteria, if applicable. | Method / Outcome measures, Exposure, Covariates, Appendix | RECORD 7.1: A complete list of codes and algorithms used to classify exposures, outcomes, confounders, and effect modifiers should be provided. If these cannot be reported, an explanation should be provided. | Method / Outcome measures, Exposure, Covariates, Appendix |  |
| Data sources/ measurement | 8 | For each variable of interest, give sources of data and details of methods of assessment (measurement).  Describe comparability of assessment methods if there is more than one group | Appendix, Methods / Outcome measures |  |  |  |
| Bias | 9 | Describe any efforts to address potential sources of bias | (Methods / Statistical Analysis, Appendix |  |  |  |
| Study size | 10 | Explain how the study size was arrived at | Study flow chart |  |  |  |
| Quantitative variables | 11 | Explain how quantitative variables were handled in the analyses. If applicable, describe which groupings were chosen, and why | Appendix |  |  |  |
| Statistical methods | 12 | (a) Describe all statistical methods, including those used to control for confounding  (b) Describe any methods used to examine subgroups and interactions  (c) Explain how missing data were addressed  (d) *Case-control study* - If applicable, explain how matching of cases and controls was addressed  (e) Describe any sensitivity analyses | (a) Methods / Statistical Analysis, Appendix  (b) Methods / Statistical Analysis  (c) Methods / Statistical Analysis  (d) Methods / Statistical Analysis , Appendix  (e)Methods / Statistical Analysis | | |  |
| Data access and cleaning methods |  | .. |  | RECORD 12.1: Authors should describe the extent to which the investigators had access to the database population used to create the study population.  RECORD 12.2: Authors should provide information on the data cleaning methods used in the study. | Methods / Data  Methods/ Data |  |
| Linkage |  | .. |  | RECORD 12.3: State whether the study included person-level, institutional-level, or other data linkage across two or more databases. The methods of linkage and methods of linkage quality evaluation should be provided. | Methods/Data |  |
| **Results** | | | | | | |
| Participants | 13 | (a) Report the numbers of individuals at each stage of the study (*e.g.*, numbers potentially eligible, examined for eligibility, confirmed eligible, included in the study, completing follow-up, and analysed)  (b) Give reasons for non-participation at each stage.  (c) Consider use of a flow diagram | Study flow chart | RECORD 13.1: Describe in detail the selection of the persons included in the study (*i.e.,* study population selection) including filtering based on data quality, data availability and linkage. The selection of included persons can be described in the text and/or by means of the study flow diagram. | Study flow chart |  |
| Descriptive data | 14 | (a) Give characteristics of study participants (*e.g.*, demographic, clinical, social) and information on exposures and potential confounders  (b) Indicate the number of participants with missing data for each variable of interest | (a, b) Table 1 |  |  |  |
| Outcome data | 15 | *Case-control study* - Report numbers in each exposure category, or summary measures of exposure  *Cross-sectional study* - Report numbers of outcome events or summary measures | Table 1 |  |  |  |
| Main results | 16 | (a) Give unadjusted estimates and, if applicable, confounder-adjusted estimates and their precision (e.g., 95% confidence interval). Make clear which confounders were adjusted for and why they were included  (b) Report category boundaries when continuous variables were categorized  (c) If relevant, consider translating estimates of relative risk into absolute risk for a meaningful time period | Table 3  Table 1  Not applicable |  |  |  |
| Other analyses | 17 | Report other analyses done—e.g., analyses of subgroups and interactions, and sensitivity analyses | Results, Appendix |  |  |  |
| **Discussion** | | | | | | |
| Key results | 18 | Summarise key results with reference to study objectives | Discussion 1^st^ Paragraph |  |  |  |
| Limitations | 19 | Discuss limitations of the study, taking into account sources of potential bias or imprecision. Discuss both direction and magnitude of any potential bias | Discussion/ limitations | RECORD 19.1: Discuss the implications of using data that were not created or collected to answer the specific research question(s). Include discussion of misclassification bias, unmeasured confounding, missing data, and changing eligibility over time, as they pertain to the study being reported. | Discussion / 1^st^ paragraph, limitations |  |
| Interpretation | 20 | Give a cautious overall interpretation of results considering objectives, limitations, multiplicity of analyses, results from similar studies, and other relevant evidence | Discussion, 1^st^ section |  |  |  |
| Generalisability | 21 | Discuss the generalisability (external validity) of the study results | Discussion, 1^st^ section |  |  |  |
| **Other Information** | | | | | | |
| Funding | 22 | Give the source of funding and the role of the funders for the present study and, if applicable, for the original study on which the present article is based | Funding section |  |  |  |
| Accessibility of protocol, raw data, and programming code |  | .. |  | RECORD 22.1: Authors should provide information on how to access any supplemental information such as the study protocol, raw data, or programming code. | Data availability |  |

*Reference: Benchimol EI, Smeeth L, Guttmann A, Harron K, Moher D, Petersen I, Sørensen HT, von Elm E, Langan SM, the RECORD Working Committee. The REporting of studies Conducted using Observational Routinely-collected health Data (RECORD) Statement. *PLoS Medicine* 2015; in press.

*Checklist is protected under Creative Commons Attribution ([CC BY](http://creativecommons.org/licenses/by/4.0/)) license.

References

1.	National Collaborating Centre for Mental Health. The Improving Access to Psychological Therapies Manual 2021 [Available from: <https://www.england.nhs.uk/wp-content/uploads/2018/06/the-iapt-manual-v5.pdf>.

2.	NHS Digital. Hospital Episode Statistics (HES) 2021 [Available from: <https://digital.nhs.uk/data-and-information/data-tools-and-services/data-services/hospital-episode-statistics>.

3.	NHS Digital. Mental Health Services Data Set 2021 [Available from: <https://digital.nhs.uk/data-and-information/data-collections-and-data-sets/data-sets/mental-health-services-data-set>.

4.	NHS Digital. Linked HES-ONS mortality data 2020 [Available from: <https://digital.nhs.uk/data-and-information/data-tools-and-services/data-services/linked-hes-ons-mortality-data>.

5.	National Collaborating Centre for Mental Health. The Improving Access to Psychological Therapies Manual. National Collaborating Centre for Mental Health; 2021 2021.

6.	Buckman JEJ, Saunders R, Stott J, Arundell LL, O'Driscoll C, Davies MR, et al. Role of age, gender and marital status in prognosis for adults with depression: An individual patient data meta-analysis*. Epidemiol Psychiatr Sci*. 2021;30:e42.

7.	Buckman JEJ, Saunders R, Stott J, Cohen ZD, Arundell L-L, Eley TC, et al. Socioeconomic Indicators of Treatment Prognosis for Adults With Depression: A Systematic Review and Individual Patient Data Meta-analysis*. JAMA Psychiatry*. 2022;79(5):406–16.

8.	Buckman JEJ, Stott J, Main N, Antonie DM, Singh S, Naqvi SA, et al. Understanding the psychological therapy treatment outcomes for young adults who are not in education, employment, or training (NEET), moderators of outcomes, and what might be done to improve them*. Psychological Medicine*. 2021:1–12.

9.	Saunders R, Buckman JEJ, Stott J, Leibowitz J, Aguirre E, John A, et al. Older adults respond better to psychological therapy than working-age adults: evidence from a large sample of mental health service attendees*. Journal of Affective Disorders*. 2021;294:85–93.

10.	Saunders R, Cape J, Leibowitz J, Aguirre E, Jena R, Cirkovic M, et al. Improvement in IAPT outcomes over time: are they driven by changes in clinical practice? *Cogn Behav Therap*. 2020;13:e16.
